# Cross-Sectional Abdominal Imaging Findings in Patients With COVID-19

**DOI:** 10.7759/cureus.9538

**Published:** 2020-08-03

**Authors:** Kaustubh Shiralkar, Naga Chinapuvvula, Daniel Ocazionez

**Affiliations:** 1 Diagnostic Radiology, University of Texas Health Science Center at Houston, Houston, USA

**Keywords:** covid 19, sars-cov-2, novel coronavirus, abdominal pain, imaging findings

## Abstract

Objective

We aimed to review and analyze cross-sectional abdominal imaging findings in a cohort of 10 patients who had tested positive for coronavirus disease 2019 (COVID-19).

Methods

This retrospective study conducted from April 1, 2020, to May 13, 2020, involved two institutions that comprised a central tertiary academic institution and multiple smaller community hospitals. We reviewed and examined cross-sectional imaging studies of patients who tested positive for COVID-19 either during the emergency room (ER) visit or hospital admission. Salient imaging findings and medical records were reviewed.

Results

A total of 10 COVID-19-positive patients (seven males and three females) of ages ranging from 21-75 years underwent cross-sectional abdominopelvic imaging. Nine of the 10 patients demonstrated typical lung base findings associated with COVID-19 on both CT and MRI. Twelve CT abdominopelvic examinations, one MRI abdomen, and one right upper quadrant ultrasound (RUQ US) were performed, with three patients undergoing two CT scans during the course of hospitalization. Gastric and bowel wall abnormalities were found on 25% (n=3/12) of abdominal CT scans. Acute interstitial pancreatitis and acute cholecystitis were both found on one CT exam. The remaining (n=7/12, 58%) CT studies demonstrated no acute intraabdominal pathology with incidental findings including fatty liver disease, cirrhosis, and splenomegaly.

Conclusion

A spectrum of abdominal imaging findings ranging from colitis to pancreatitis may be correlated with COVID-19 infection, even though the majority of our patients with gastrointestinal (GI) symptoms did not have identifiable GI pathology on imaging.

## Introduction

Information regarding coronavirus disease 2019 (COVID-19) is constantly evolving and clinical presentations besides fever and respiratory tract symptoms are being increasingly recognized. Severe acute respiratory syndrome coronavirus 2 (SARS-CoV-2), the virus that causes COVID-19, is thought to have originated in Wuhan, China in December 2019 and is now the cause of an ongoing and historical pandemic. The SARS-CoV-2 gains entry into human cells via angiotensin-converting enzyme 2 (ACE2) receptors, which are found predominately in type II pneumocytes. However, ACE2 is expressed broadly in the human body viscera [1]. These may result in a wide range of direct targets for the virus, including bowel, vasculature, liver, kidneys, heart, and brain, indicating a broad organotropism [2-4]. Hence it is no surprise that a sizeable subset of patients may initially present with and/or develop atypical clinical presentations that may be seen on imaging and affect the disease course.

Recent studies have demonstrated abdominal imaging findings that help to understand the varied imaging appearances that may be seen with disseminated or atypical infection [5,6]. Radiologists should thus be aware of potential abdominal imaging findings in patients with COVID-19. The purpose of this study was to retrospectively review and describe cross-sectional abdominal imaging findings in a series of patients with COVID-19 who were recently treated at our multi-center institution.

## Materials and methods

We conducted this retrospective study from April 1, 2020, to May 13, 2020. It involved two institutions that consist of a central tertiary academic institution and multiple smaller community hospitals. The institutional review board approved this multi-center study and written informed consent was waived. All data were collected in compliance with the Health Insurance Portability and Accountability Act (HIPAA).

We performed a retrospective search on a database (Primordial Design; Nuance Communications, Burlington, MA) linked to our picture archiving and communication systems (PACS), relating to all adult patients (>18 years) who underwent cross-sectional abdominopelvic imaging (CT, MRI, and/or US) over a six-week period (April 1, 2020, to May 13, 2020) and also tested positive for COVID-19. All patients underwent COVID-19 testing with reverse transcription-polymerase chain reaction (RT-PCR) either during the emergency room (ER) visit or hospital admission.

The CT scans were performed with (n=8) or without (n=4) intravenous (IV) contrast media on either a 64-slice or 128-slice multidetector CT scanner. In patients undergoing contrast-enhanced CT, axial acquisition of the abdomen and pelvis in the portal venous phase was performed after the injection of 80-120 cc of iodinated contrast media (Omnipaque 350 mgI/ml or Visipaque 320 mg/ml; GE Healthcare, Chicago, IL) at a flow rate of 3-5 ml/sec. Axial images were reconstructed at 5-mm thickness with an interval of 5 mm. Coronal and sagittal reformatted images were created at 3-mm thickness. 

Our US examination (n=1) was performed by utilizing a typical right upper quadrant ultrasound (RUQ US) protocol that included cine and static image evaluation of the liver, gallbladder, central biliary tree, and portal vein. The abdominal MRI examination (n=1) involved a pre- and post-contrast multiphasic liver MRI protocol with gadolinium performed on a 3 Tesla magnet.

Radiology reports and images were retrospectively reviewed by an abdominal and a thoracic radiologist with four and six years of post-fellowship experience, respectively. Abdominal and lung base findings were recorded. Clinical indications and additional important findings from the report were also recorded. The COVID-19 test results in the electronic medical records of the patients were recorded to serve as the reference standard. The electronic medical records were also reviewed to determine the symptoms the patient presented with and developed, if any, during the course of the admission and whether they required hospitalization.

## Results

A total of 10 COVID-19-positive patients (seven males and three females) of ages ranging from 21-75 years underwent cross-sectional abdominopelvic imaging (Table 1). Nine of the 10 patients demonstrated typical lung base findings associated with COVID-19 on both CT and MRI. Twelve CT abdominopelvic examinations, one MRI abdomen, and one RUQ US were performed with three patients undergoing two CT scans during their hospitalization. Eight of the 12 CTs and the MRI of the abdomen were performed with IV contrast. Clinical indications of CT imaging primarily included abdominal pain (n=6/12, 50%) and nausea, vomiting, or diarrhea (n=4/12, 33%). Other clinical indications included abdominal distension and gastrointestinal (GI) bleed. The indication for the RUQ US was right upper quadrant pain and the MRI liver for liver derangement.

**Figure 1:**
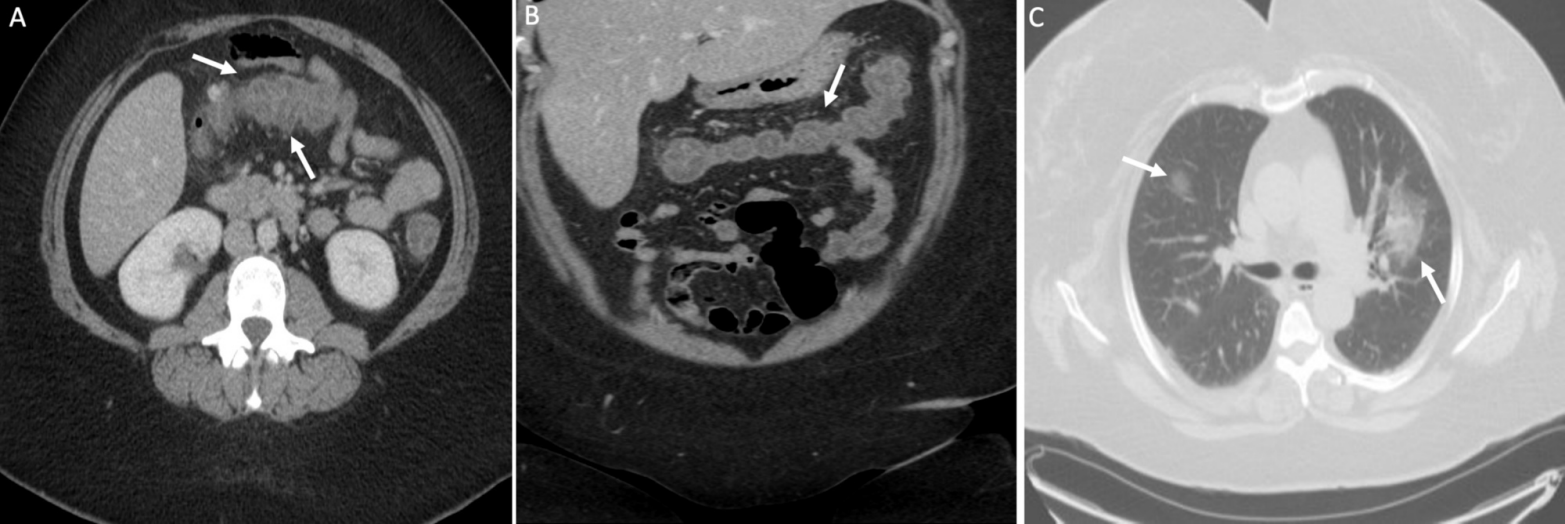
CT showing acute colitis Axial (A), coronal (B), and axial (C) CT of the abdomen and pelvis with IV contrast of a 24-year-old woman with abdominal pain and diarrhea. The edematous appearance of the transverse colon (arrows) with surrounding hyperemia is compatible with acute colitis. Lung findings in image C show typical COVID-19-associated bilateral nodular ground-glass opacities (arrows) CT: computed tomography; IV: intravenous; COVID-19: coronavirus disease 2019

**Figure 2:**
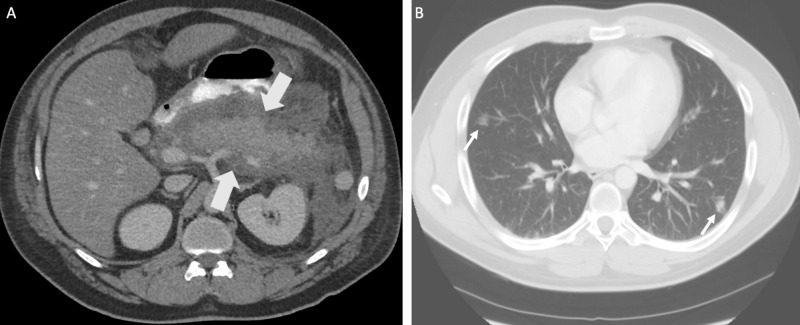
CT showing acute pancreatitis Axial (A) and (B) CT of the abdomen and pelvis with IV contrast of a 45-year-old man with abdominal pain. There is enlargement and edema of the pancreas with surrounding fluid and stranding (arrows), compatible with acute edematous pancreatitis. Ill-defined ground-glass opacities are also seen at lung bases (thin arrows in B) CT: computed tomography; IV: intravenous

Most CT scans were performed with IV contrast (n=8/12, 67%). Gastric and bowel wall abnormalities were found on 25% (n=3/12) of abdominal CT scans (Figure 1). Acute interstitial pancreatitis and acute cholecystitis were both found on one CT exam (Figure 2). The remaining (n=7/12, 58%) CT studies demonstrated no acute intraabdominal pathology with incidental findings including fatty liver disease, cirrhosis, and splenomegaly. Both the contrast-enhanced MRI and RUQ US demonstrated no acute abnormalities. Table 2 briefly summarizes the findings seen in abdominopelvic imaging by modality.

**Table 1:** Patient demographics and indications for abdominal imaging SD: standard deviation; CT: computed tomography; MRI: magnetic resonance imaging; US: ultrasound; GI: gastrointestinal; LFT: liver function test

Characteristic or indication	Value
Age (years)	
· Range	21-75
· Mean ±SD	45.1 ±19.6
Sex, n	
· Male	7
· Female	3
Clinical indication for imaging (includes CT, MRI, and US), n	
· Abdominal pain	6
· Diarrhea	4
· Nausea or vomiting	3
· Abdominal distension	2
· GI bleed	2
· Abnormal LFTs	1

**Table 2:** Descriptive data of ER and admitted patients CT: computed tomography; RUQ US: right upper quadrant ultrasound; MRI: magnetic resonance imaging; ER: emergency room

Variables	All	Discharged from ER	Admitted to hospital
Number of patients	10	3	7
Number of CT abdomen and pelvic studies	12
· No acute findings	7
· Colitis	2
· Gastritis	1
· Acute pancreatitis	1
· Acute cholecystitis	1
RUQ US	1 total		
· No acute findings	1		
MRI abdomen	1 total		
· No acute findings	1		

## Discussion

Non-productive cough, shortness of breath, and fever are the most common clinical presenting symptoms of patients with COVID-19. Abdominal signs and symptoms are being increasingly reported as familiarity with COVID-19 grows, ranging from liver enzyme elevation to bowel necrosis. Viral detection in biopsy specimens and stool provide explanations for the GI symptoms, potential recurrence, and transmission [7]. These are postulated to be related to the virus's use of ACE2 receptors located on certain intestinal cells, cholangiocytes, and hepatocytes. In addition, a significant proportion of COVID-19 patients can present initially with only digestive complaints, with the most common symptoms being anorexia, nausea, vomiting, and diarrhea. Liver-related transaminases are also elevated in a substantial proportion of patients, although generally only mildly elevated. Our series demonstrated generalized abdominal pain and nausea, vomiting, and diarrhea to be the most common indications for abdominal imaging, accounting for the majority of CT imaging indications (10/12, 83%).

Although nearly half of all patients with COVID-19 may initially present with digestive symptoms, a small number of patients only develop isolated abdominal signs and symptoms [8]. Seven out of the 10 patients in our series initially presented to ER with GI complaints, while the other three developed such signs or symptoms during admission.

Respiratory parenchymal injury may also precede symptomatology; therefore, unsuspected coronavirus disease may be strongly suggested on the basis of lung findings alone on abdominopelvic CT [9,10]. Indeed, in our study, nine out of 10 patients demonstrated the typical peripheral ground-glass nodular opacities at the lung bases. This is of critical importance as the radiologist may be the first to suggest unsuspected COVID-19 on the basis of imaging findings alone.

In our study, contrast-enhanced CT was the most frequently performed imaging modality, with the most common indications being abdominal pain and nausea, vomiting, or diarrhea. Although no acute or significant imaging abnormality was predominantly seen, gastric or bowel abnormalities were seen in 25% of patients. Additional imaging findings included acute pancreatitis and acute cholecystitis. These findings are not surprising given that the ACE2 receptor to which SARS-CoV-2 binds are found in the GI tract. Bhayana et al. have also demonstrated a spectrum of abdominopelvic imaging findings that correlate with COVID-19 infection with the predominant imaging findings of bowel wall thickening and cholestasis [5].

Our series illustrates that the clinical presentation of COVID-19 encompasses diverse and atypical presentations often involving the GI tract, and clinicians and radiologists should be aware of this spectrum. Abdominal and back pain has long been described as a symptom of pneumonia in both adults and children and is thought to be secondary to pleural irritation [11,12]. This represents an additional mechanism by which underlying lung injury can be associated with GI symptoms.

The limitations of the study include its retrospective design and small sample size. Also, we have no way of showing if the symptomatology and imaging findings were directly caused by COVID-19. Unless the SARS-CoV-2 virus can be detected in the afflicted tissues, the abdominal manifestations could be an effect of the COVID-19 disease and may not have been caused by the virus itself. In addition, pathologic correlation and clinical follow-up were not available for many patients with imaging abnormalities.

## Conclusions

Our series showed that a spectrum of abdominal imaging findings may be correlated with SARS-CoV-2 infection, even though the majority of our patients with GI symptoms did not have identifiable GI pathology on imaging. As COVID-19 testing becomes more widely accessible, it can be employed more readily in patients with atypical symptoms and abdominal complaints. This may lead to more prompt diagnosis, contact tracing, and quarantine, which would be of benefit to both the patients and public health in general.
